# Endoscopic ultrasound-guided coil and glue treatment for gastroduodenal artery pseudoaneurysm

**DOI:** 10.1055/a-2493-3544

**Published:** 2024-12-19

**Authors:** Lu Hao, Yiting Huang, Qiang Huang, Fenming Zhang, Guoqiang Xu, Hongtan Chen

**Affiliations:** 1 71069 Department of Gastroenterology, The First Affiliated Hospital of Zhejiang University School of Medicine, Hangzhou, China; 2 71069 Department of Radiology, The First Affiliated Hospital of Zhejiang University School of Medicine, Hangzhou, China

Gastroduodenal artery pseudoaneurysm (GDAP) is usually treated by transcatheter arterial embolization (TAE) 1 . However, when the pseudoaneurysm combines with an abnormal course of the artery, TAE may fail. Herein, we report a case in which endoscopic ultrasound-guided coil and glue injection (EUS-CGI) was useful for treatment of a GDAP.

A 58-year-old man with a GDAP underwent TAE prior to presentation to our hospital. The TAE failed due to the variant course of a communicating artery ( Fig. 1 ). An ultrasound endoscope was introduced, and a 22-gauge puncture needle (EchoTip, Ultra Endoscopic Ultrasound Needle; Cook Medical, Bloomington, Indiana, USA) was selected to penetrate the GDAP. A preloaded 0.018-inch fibered interlock detachable coil (F-IDC; 2D 12 mm, 30 cm; Boston Scientific, Marlborough, Massachusetts, USA) and two 0.18-inch pushable coils (2D 12 mm, 5 cm; Boston Science, Marlborough, Massachusetts, USA) were slowly pushed out of the needle tip into the entrance of the GDAP. Thereafter, 2 ml saline followed by 0.5 ml glue and 2 ml saline was injected rapidly. Subsequent color Doppler imaging showed that the blood flow had disappeared ( Video 1 ).

**Fig. 1:**
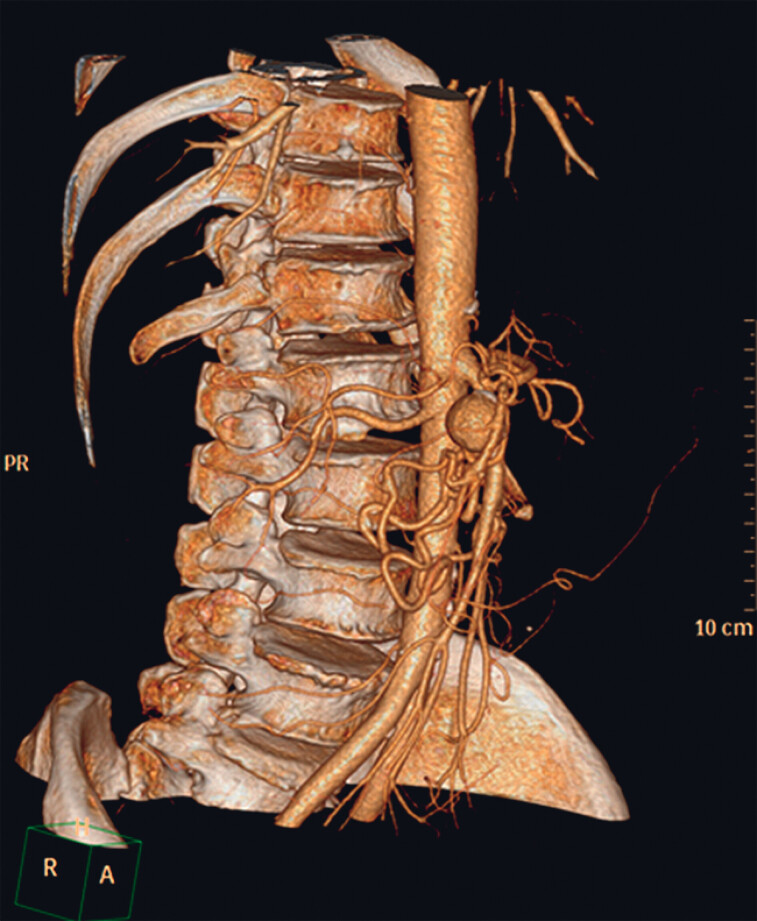
Gastroduodenal artery pseudoaneurysm (GDAP) shown by gastric vascular CT angiography (CTA).

Endoscopic ultrasound-guided coil and glue treatment for a gastroduodenal artery pseudoaneurysm.Video 1

Gastric arterial CT angiography (CTA) was performed for follow-up and revealed the GDPA to be partially filled with contrast ( Fig. 2 ). The patient was therefore readmitted. Under EUS, the GDAP showed a hyperechoic mass with posterior acoustic shadow. Flows could be seen in the residual cavity, and pulsed wave Doppler imaging showed arterial blood flow, indicating recanalization. The residual cavity was punctured with a 22-gauge needle. Two 0.18-inch pushable coils were slowly placed. Thereafter, 2 ml saline followed by 0.5 ml glue and 2 ml saline was injected rapidly. Doppler imaging after the intervention showed the flow to have disappeared ( Video 1 ). Gastric arterial CTA was performed 2 days later and showed no obvious contrast filling ( Fig. 3 ).

**Fig. 2:**
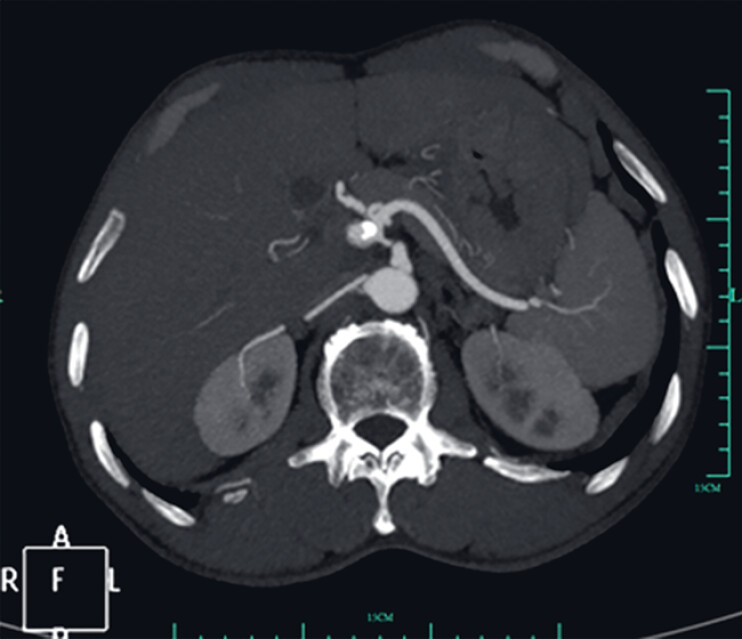
Gastric artery CTA after the first endoscopic ultrasound (EUS) intervention.

**Fig. 3:**
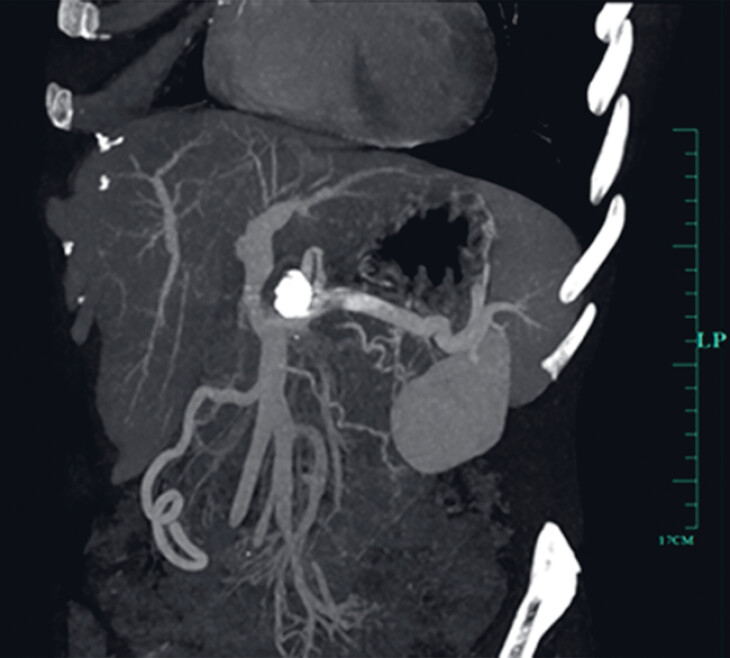
Gastric artery CTA after the EUS second intervention showed the GDAP to be completely embolized.

EUS-CGI as a treatment for GDAP makes use of the natural lumen of the digestive tract. It is not only a safe and effective treatment for GDAP, but also is repeatable, accurate, and avoids radiation exposure.

Endoscopy_UCTN_Code_TTT_1AS_2AG

## Notes

Endoscopy E-Videos https://eref.thieme.de/e-videos
*E-Videos* is an open access online section of the journal *Endoscopy* , reporting on interesting cases and new techniques in gastroenterological endoscopy. All papers include a high-quality video and are published with a Creative Commons CC-BY license. Endoscopy E-Videos qualify for HINARI discounts and waivers and eligibility is automatically checked during the submission process. We grant 100% waivers to articles whose corresponding authors are based in Group A countries and 50% waivers to those who are based in Group B countries as classified by Research4Life (see: https://www.research4life.org/access/eligibility/ ). This section has its own submission website at https://mc.manuscriptcentral.com/e-videos .
